# Acute inspiratory resistance training enhances endothelium‐dependent dilation and retrograde shear rate in healthy young adults

**DOI:** 10.14814/phy2.15943

**Published:** 2024-02-04

**Authors:** Dallin Tavoian, Josie L. Mazzone, Daniel H. Craighead, E. Fiona Bailey

**Affiliations:** ^1^ Arizona Respiratory Neurophysiology Laboratory, Department of Physiology University of Arizona Tucson Arizona USA; ^2^ Department of Integrative Physiology University of Colorado Boulder Boulder Colorado USA

**Keywords:** endothelial, exercise, flow‐mediated dilation, respiratory, shear rate

## Abstract

Inspiratory resistance training (IRT) yields significant reductions in resting blood pressure and improves vascular endothelial function. Our objective was to quantify the acute effects of IRT on brachial artery flow‐mediated dilation (FMD) and shear rates (SRs) in healthy men and women. Twenty young adults (22.9 ± 3.4 years; 10 male, 10 female) completed a single bout of IRT or Rest condition in a randomized crossover design. Brachial artery FMD was performed before, 10 min after, and 40 min after the assigned condition. Brachial artery blood flow velocities were collected during IRT, separated by breathing cycle phase, and converted into SRs. FMD improved 10 min post‐IRT (+1.86 ± 0.61%; *p* = 0.025) but returned to baseline by 40 min post‐IRT (*p* = 0.002). Anterograde SR decreased by 10% and retrograde SR increased 102% during resisted inspiration, relative to baseline SR (*p* < 0.001). Anterograde SR increased by 7% in men and women (*p* < 0.001) and retrograde SR decreased by 12% in women but not men (*p* = 0.022) during unresisted expiration, relative to baseline SR. A single bout of IRT elicits a transient enhancement in FMD in both men and women. Acute IRT‐related enhancements in SRs may contribute to sustained improvements in FMD that have been reported previously.

## 1 INTRODUCTION

Unfavorable vascular remodeling (e.g., increased arterial stiffness and endothelial dysfunction) is a byproduct of aging and sedentary behavior (Renna et al., 2013). Regular exercise can prevent this remodeling, and even reverse it once established (Parker et al., 2010; Prior et al., 2003). For example, flow‐mediated dilation (FMD), the standard for noninvasive assessment of conduit artery endothelial function (Deanfield et al., 2007), improves in response to exercise, secondary to enhanced nitric oxide production and bioavailability and reduced oxidative stress (Gielen et al., 2010; Higashi & Yoshizumi, 2004). Acutely, exercise increases blood flow, and so increases the shear stress applied to the endothelial cells (Lyall et al., 2019; Tinken et al., 2010), an effect believed to be the primary mechanism underlying exercise‐induced vascular remodeling (Tinken et al., 2010). Specifically, exercise greatly increases anterograde (i.e., laminar) shear rate (SR) without substantially increasing retrograde (i.e., reverse) SR, resulting in elevated mean SR (mean SR = anterograde SR − retrograde SR) for the duration of exercise (Green et al., 2017). While the acute and long‐term effects of traditional aerobic exercise on SR have been described in detail (Green et al., 2017), the effects of less conventional respiratory‐type training interventions on vascular health and SRs are unknown.

Inspiratory resistance training (IRT) is a resisted breathing exercise initially developed to improve respiratory strength and endurance (Leith & Bradley, 1976). In 2015, Vranish and colleagues (Vranish & Bailey, 2015) discovered resting blood pressure reduction was an unexpected byproduct of regular IRT performance, the result of repeated generation of large negative intrathoracic pressures. Subsequent studies have shown that 6 weeks of daily IRT lowers systolic and diastolic blood pressures by 9 and 4 mmHg, respectively, in normotensive and hypertensive adults (Craighead et al., 2022), and improves brachial artery FMD by 45% in generally healthy older adults with above‐normal blood pressure (Craighead et al., 2021). Notably, IRT is distinct from traditional forms of exercise in several regards. First, IRT comprises five sets of six breaths against a resistance set at 75% of an individual's maximal inspiratory strength and a total training time of just 5–10 min each day (Craighead et al., 2022). Second, it does not require activation of large (limb) muscle groups, and so changes in blood flow are not likely due to increased metabolic demand. Finally, with minimal equipment demands it can be performed anywhere.

IRT is a time‐efficient, non‐pharmacological exercise with clear potential as an adjunctive therapy for adults with elevated cardiovascular disease risk; however, the mechanisms underlying IRT‐related improvements in blood pressure and endothelial function are unknown. Our objective was to evaluate the effects of a single bout of IRT on brachial artery FMD and carotid‐femoral pulse wave velocity (PWV) using a within‐subject crossover design. We also measured brachial artery SR patterns (anterograde, retrograde, and mean SR) and diameter during and after IRT to elaborate on the relationship between shear variables and acute IRT‐induced changes in FMD. Although we have not previously observed sex‐specific differences in blood pressure or endothelial function in response to 6 weeks of IRT (Craighead et al., 2021, 2022), we considered sex as a factor in the current analyses. We hypothesized FMD would be greater following IRT, relative to baseline, and that the change in FMD would be associated with the mean SR. Finally, we did not expect a single bout of IRT would affect arterial stiffness (i.e., PWV).

## 2 METHODOLOGY

### 2.1 Subjects

Twenty healthy young adults (22.9 ± 3.4 years; 10 men, 10 women) were recruited to examine the short‐term effects of a single bout of IRT on brachial artery endothelial function and SRs, as well as arterial stiffness. All participants (1) were free of cardiovascular, neurological, and respiratory diseases, (2) had a body mass index <40 kg/m^2^, (3) did not have asthma, (4) were non‐smokers, and (5) did not have a history of collapsed lung, perforated eardrum, or detached retina (see Table 1 for participant characteristics). The study was approved by the University of Arizona institutional review board, in accordance with the Declaration of Helsinki. Written informed consent was obtained from all participants prior to data collection.

**TABLE 1:** Baseline characteristics.

	Female	Male	*p*‐value	Combined
Sample size (*n*)	10	10	–	20
Age (years)	20.6 ± 1.4	25.2 ± 3.3*	**0.002**	22.9 ± 3.4
Mass (kg)	67.3 ± 15.8	81.5 ± 13.2*	**0.043**	74.4 ± 15.9
BMI (kg/m^2^)	23.9 ± 4.7	24.8 ± 3.2	0.623	24.3 ± 3.9
SBP (mmHg)	115.4 ± 9.7	126.2 ± 12.3*	**0.043**	120.8 ± 12.2
DBP (mmHg)	61.6 ± 4.4	65.5 ± 10.1	0.281	63.6 ± 7.9
MAP (mmHg)	79.5 ± 5.1	85.7 ± 10.3	0.110	82.6 ± 8.5
Absolute FMD (mm)	0.29 ± 0.07	0.18 ± 0.07*	**0.003**	0.24 ± 0.09
Relative FMD (%)	9.76 ± 2.35	4.57 ± 1.76*	**<0.001**	7.17 ± 3.34
Shear normalized FMD (%)	4.79 ± 1.02	3.00 ± 1.04*	**0.001**	3.90 ± 1.36
Scaled FMD (%)	9.73 ± 2.15	4.30 ± 2.05*	**0.003**	6.98 ± 3.35
PWV (m/s)	6.33 ± 0.83	7.62 ± 0.86*	**0.004**	6.94 ± 1.06
PI_MAX_ (cmH_2_O)	−86.0 ± 12.7	−105.1 ± 21.5*	**0.029**	−95.6 ± 19.7
Training load (cmH_2_O)	−43.1 ± 6.3	−52.3 ± 10.9*	**0.036**	−47.7 ± 9.8
Training power (W)	5.17 ± 2.19	8.87 ± 2.10*	**0.001**	7.02 ± 2.82
Training volume (L)	2.16 ± 0.98	3.49 ± 0.41*	**<0.001**	2.83 ± 1.00

*Note*: Data are mean ± SD. Unpaired *t*‐tests; male versus female. Bold text indicates significant *p*‐value.

Abbreviations: BMI, body mass index; DBP, diastolic blood pressure; FMD, flow‐mediated dilation; MAP, mean arterial pressure; PI_MAX_, maximal inspiratory pressure; PWV, pulse wave velocity; SBP, systolic blood pressure.

* Males significantly different than females, *p* < 0.05.

### 2.2 Study design

Participants reported to the laboratory three times for this within‐subjects crossover study, with each visit separated by at least 48 h. We did not control for menstrual cycle phase in female participants so as to permit generalizability of the results (Stanhewicz & Wong, 2020). The initial visit comprised informed consent, health screening, height and weight assessment, inspiratory strength assessment, and familiarization with the IRT protocol. For the subsequent experimental visit, participants were block randomized to complete a single bout of IRT or a time‐matched Rest (i.e., control) condition. For the final visit, each participant completed the opposite protocol to that completed during their previous experimental visit. The block randomization allocation sequence was stratified by participant sex and balanced so that half of the participants completed IRT first, while the other half completed the Rest condition first. The experimental visits were identical, with the exception of the IRT or Rest condition being completed that day. Participants arrived at the laboratory following abstinence from exercise, alcohol, and caffeine for >24 h, and >5h fasting.

**FIGURE 1:**
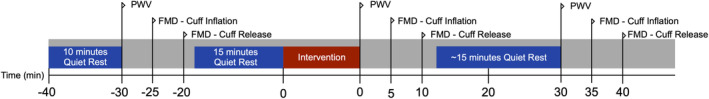
Experimental timeline.

Participants rested for 10 min before baseline arterial stiffness and FMD recordings. All measurements were performed with participants in supine. Brachial artery blood pressure was recorded every 5 min using an automated oscillometric blood pressure machine (SunTech CT40, Suntech Medical, Morrisville, NC, USA) on the arm not used for FMD assessment. After baseline PWV and FMD recordings were completed, participants continued to rest in supine for an additional 15 min. At the end of the 15 min, participants completed either the IRT or Rest condition (described below), immediately followed by measures of PWV. FMD measurement was started ~3 min post‐IRT/Rest. Participants then rested quietly until 30 min post‐IRT/Rest, at which point PWV was repeated. FMD was started ~33 min post‐IRT/‐Rest (Figure 1).

### 2.3 Experimental protocol

#### 2.3.1 Inspiratory strength assessment

Maximal inspiratory pressure (PI_MAX_) measured at the mouth was assessed with a Hans Rudolph non‐rebreathing device (2600 series; Hans Rudolph, Shawnee, KS, USA), in accordance with the American Thoracic Society and European Respiratory Society recommendations (ATS/ERS statement on respiratory muscle testing, 2002). The device has a two‐way non‐rebreathing valve. The inspiration port was blocked with an endcap with a small hole (~2 mm diameter) to allow a small amount of airflow during inspiration to prevent glottal closure, providing constant, near‐maximal resistance. The expiration port opened to the room, providing no resistance during expiration. In a supine position, participants applied nose plugs and were instructed to place the flanged mouthpiece in their mouth, not allowing space between the mouthpiece and their lips for air to escape or enter. They were then instructed to empty their lungs completely, and then inhale as quickly and forcefully as possible for at least 2 s. Participants were allowed to practice the PI_MAX_ maneuver up to 20 times, with at least 15 s of rest between efforts. Pressure tracings were monitored to ensure the maneuver was performed correctly. The participant then performed the PI_MAX_ maneuver until three attempts fell within 10% of each other (maximum of eight attempts), and those three values were averaged and used for analysis.

#### 2.3.2 Inspiratory resistance training condition

Participants completed a single bout of IRT using a POWERbreathe K3 Trainer (POWERbreathe International, Warwickshire, UK), an electronic threshold resistance device, while in supine with their right arm abducted to ~90° for ultrasound assessment of brachial artery blood flow. Target pressure was set at 50% of participants' supine PI_MAX_. Note that the standardized IRT protocol calls for inspiratory resistance to be set at 75% of PI_MAX_; however, most naïve participants have difficulty performing IRT at 75% of PI_MAX_, and initial training levels typically are set at a lower relative resistance (i.e., 50%–55%) to ensure correct technique. The training protocol consisted of five sets of six breaths against the target resistance at a breathing rate of ~8 breaths/min (0.13 Hz, duty cycle 1:2 [inspiration:expiration]), with 60 s rest between sets, for a total training time of ~8 min. Pacing of breaths during each interval of IRT was determined by the K3 training device. Participants wore nose plugs during the training bout.

#### 2.3.3 Rest condition

The Rest condition consisted of 8 min of quiet rest in a supine position with the participant's right arm abducted to ~90° for ultrasound assessment of brachial artery blood flow. Participants were instructed to rest quietly for 8 min. No guidance was provided regarding breathing rate, depth, or duty cycle.

### 2.4 Experimental measures

#### 2.4.1 Baseline characteristics

Participant characteristics at baseline are reported in Table 1. FMD and PWV variables from pre‐IRT and pre‐Rest visits were averaged and used for analysis.

#### 2.4.2 Flow‐mediated dilation and blood flow velocity

Imaging of the brachial artery was performed using high‐resolution ultrasonography (Canon Xario 200G, Canon Medical Systems, Tustin, CA, USA). The same investigator performed all scans. Participants were supine with their right arm abducted to ~90° and a rapid inflation/deflation pneumatic cuff was applied to the forearm ~3 cm distal to the medial humoral epicondyle. A multifrequency linear array probe was used to image the brachial artery in the distal half of the upper right arm, proximal to the pneumatic cuff. When an optimal image was obtained, a multi‐jointed support arm was used to assist the investigator in holding the probe in place. Unique anatomical landmarks within the image were identified, probe location was marked on the skin with permanent marker, and probe distance from the antecubital fossa was recorded to ensure identical placement for repeat measurements. Continuous Doppler velocity images were obtained using the ultrasound at a 60° insonationangle to quantify blood flow velocity. Images were collected and analyzed using the Vascular Research Tools version 6.7.4 (Vascular Imager, MIA, Coralville, IA, USA), which uses a participant's electrocardiograph (ECG) signal to trigger image collection during diastole. All ultrasound settings were kept the same for individual participants during each FMD recording.

Baseline brachial artery diameter and flow velocity were recorded for 1 min, after which the pneumatic cuff was inflated to ~200 mmHg (>50 mmHg higher than systolic blood pressure for all participants) for 5 min. Diameter and flow recordings resumed 30 s prior to cuff deflation and continued for 3 min after cuff deflation, following current recommendations (Thijssen et al., 2019).

#### 2.4.3 Brachial artery diameter and blood flow velocity during IRT

Brachial artery diameter and flow velocity were collected continuously during the IRT condition at the same site on the upper arm where pre/post‐assessments were completed. Recording began 30 s prior to the start of the assigned condition and ended 2 min post‐intervention. As with FMD, the ECG signal served as the trigger for image collection. Anatomical landmarks within the image were identified to ensure relative position of the artery was maintained on screen.

#### 2.4.4 Pulse wave velocity

Arterial stiffness was assessed via carotid‐femoral PWV using a transcutaneous tonometer (SPT‐301 AD Instruments, Colorado Springs, CO, USA). Pulse pressure was measured over the skin at the carotid artery for ~1 min, followed by the femoral artery for ~1 min. Electrical heart rhythm was recorded simultaneously with a three‐lead ECG. Data were sampled at 1000 Hz.

#### 2.4.5 Training outcomes

The POWERbreathe K3 training device has an onboard computer that automatically records and averages breathing power and volume for each training session. Breathing power is a measure of inspiratory force and velocity, measured in watts (W). Volume is the amount of air inspired per breath, measured in liters.

### 2.5 Data analysis

#### 2.5.1 Flow‐mediated dilation and hyperemic shear rate

Brachial artery diameter was assessed offline using commercial automated edge detection software (Brachial Analyzer for Research, MIA, Coralville, IA, USA). Resting diameter was determined as mean diameter during the 1‐min baseline recording, peak diameter was the greatest diameter recorded following cuff deflation. The formula for relative FMD is:

$$\begin{array}{c} \text{Relative}\;\mathrm{FMD}\;\left(\%\right)=\\ \;\left(\text{peak diameter}-\text{resting diameter}\right)/\text{resting diameter}\times 100 \end{array}$$

Resting flow velocity was calculated as the 10‐pulse average of peak velocity during the baseline recording. Hyperemia was calculated as the 10‐pulse average of peak velocity immediately following cuff deflation. Velocity then was converted to SR with the following formula (Ghardashi Afousi et al., 2018):

$$\mathrm{SR}\;\left(s^{-1}\right)=\left(\text{blood velocity}/\text{resting diameter}\right)\times 4$$

#### 2.5.2 Brachial artery diameter and shear rate during IRT

Pre‐intervention (i.e., baseline) flow velocity was calculated as the 30 s average of peak velocity of each pulse immediately before the start of IRT. During IRT, flow velocity was quantified separately for inspiration and expiration phases of the breathing cycle. Peak anterograde and retrograde flow velocity from 18 efforts (first and last set of efforts were excluded) were averaged and SRs were calculated using the formula referenced previously. The first two pulses following the initiation of inspiration and the first four pulses following the initiation of expiration were used for the analysis, matching the 1:2 duty cycle of the breathing efforts. Arterial diameter was determined during each of the 1‐min rest intervals between sets.

#### 2.5.3 Allometric scaling and shear normalization

It is well known that there is a negative correlation between relative FMD and baseline arterial diameter, and thus scaling relative FMD to account for differences between groups is sometimes necessary (Atkinson & Batterham, 2013). Allometric scaling was not necessary in our repeated measures design, with the exception of sex difference analyses. Thus, for assessments of sex differences, we performed statistical tests on scaled and unscaled data. Any differences in the results of the scaled and unscaled analyses are noted in the results.

We also controlled for differences in the hyperemic response to the 5‐min cuff inflation protocol (i.e., shear stimulus). The formula for shear normalized FMD is:

$$\begin{array}{c} \text{Shear normalized}\;\mathrm{FMD}=\\ \;\left(\text{relative}\;\mathrm{FMD}/\text{hyperemic shear rate}\right)\times 100 \end{array}$$

Any differences between the shear normalized and relative FMD analyses are noted in the results.

#### 2.5.4 Pulse wave velocity

Transit time was defined as time difference(s) between the ECG R‐wave and foot of the pulse pressure wave (i.e., second derivative of the pulse wave). Transit time was calculated and averaged over 30 carotid and 30 femoral pulses. Distance (cm) between the carotid and femoral measurement sites was measured and multiplied by 0.8, as recommended by expert consensus (Van Bortel et al., 2012). The formula for PWV is:

$$\mathrm{PWV}=\left(d\times 0.8\right)/\left({\mathrm{TT}}_{\mathrm{FEM}}-{\mathrm{TT}}_{\mathrm{CAR}}\right)$$

where *d* = distance (*m*), TT_FEM_ = femoral transit time (s), and TT_CAR_ = carotid transit time (s). PWV data from one male participant were sampled at a rate that was too low for accurate analysis (i.e., 10 Hz); thus, results are reported for (*n* = 19) for this measure.

### 2.6 Statistical analysis

Statistical analysis was completed using SPSS Statistics v28 (IBM, Chicago, IL, USA). Pre–post outcome variables were compared with a two‐way repeated measures ANOVA (condition × sex × time) for between‐ and within‐day analyses. Outcomes collected during IRT were compared with repeated measures ANOVAs (sex × time). Significant main effects and interactions were followed with Sidak post hoc analyses. Sex differences in baseline characteristics were assessed with independent *t*‐tests. Pearson correlations were performed between SR outcomes and change in FMD post‐intervention. Values are mean ± SD.

For this preliminary study we estimated a modest effect size of 0.40. We performed a power analysis using G*Power 3.1 (Faul et al., 2007) using the ANOVA: Repeated measures, within‐between interaction *F*‐test. A sample size of 12–18 would achieve 80%–95% power, and we recruited 20 participants to account for potential dropout. Our previous work has shown that there are no sex differences in response to 6 weeks of IRT, and we did not consider sex as a factor in the power analysis.

## 3 RESULTS

### 3.1 Baseline characteristics

Differences in several anthropometric and physiological outcomes were apparent between male and female participants at baseline, including age, body mass, systolic blood pressure, PI_MAX_, FMD (absolute, relative, scaled, and shear normalized), and PWV (all *p* < 0.05; Table 1). There were no between group differences in body mass index, diastolic blood pressure, or mean arterial pressure.

### 3.2 Brachial artery flow‐mediated dilation

**FIGURE 2:**
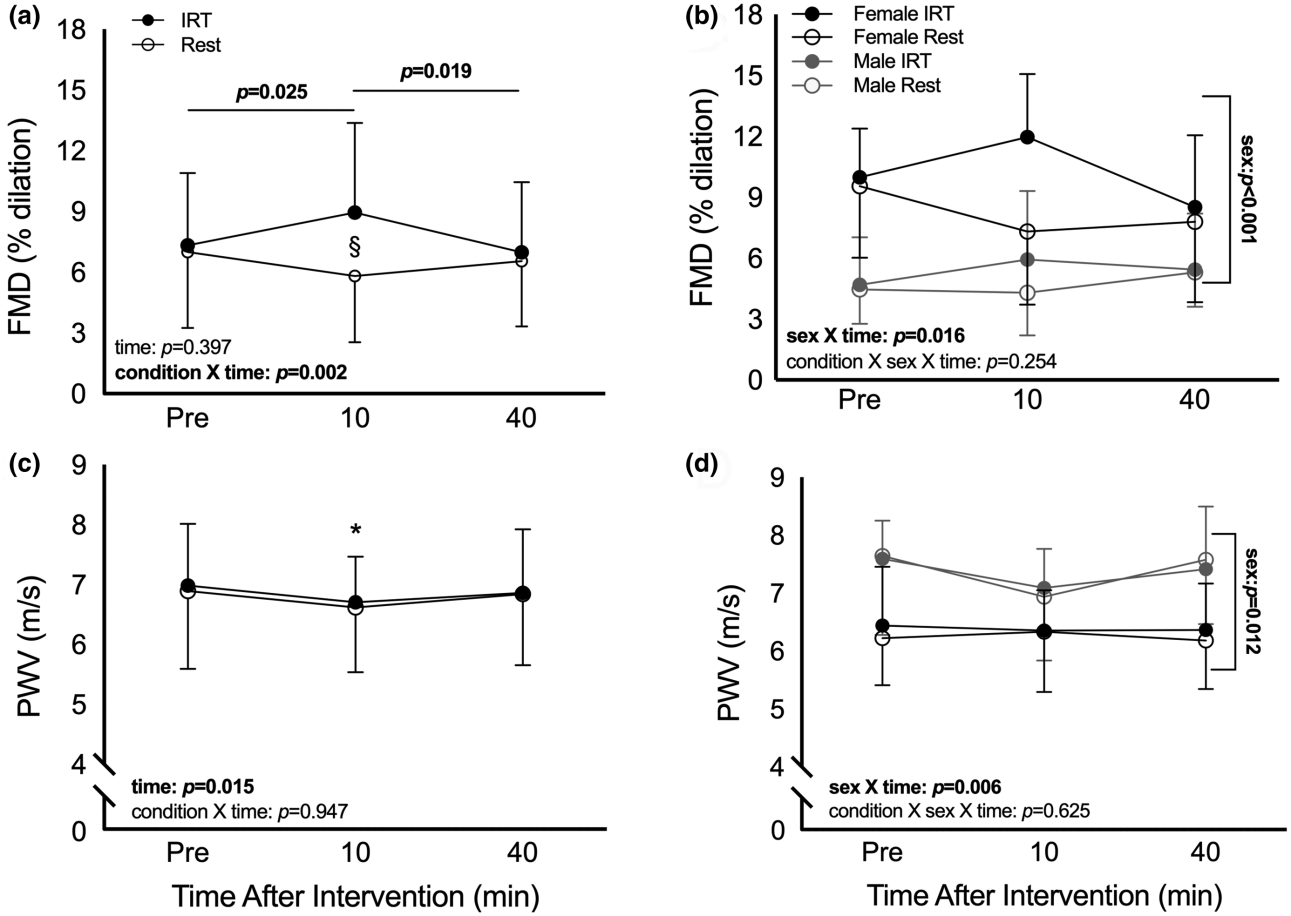
Change in relative FMD before (pre), 10 min after, and 40 min after IRT and time‐matched rest condition in healthy young men and women (a) combined (*n* = 20) and (b) separated by sex (10 male, 10 female). Change in PWV before (pre), 10 min after, and 40 min after IRT and time‐matched rest in healthy young men and women (c) combined (*n* = 19) and (d) separated by sex (9 male, 10 female). Error bars represent SD. Two‐way repeated measures ANOVA. Post hoc analyses: *main effect of time, significantly different from pre‐IRT; ^§^significantly different between interventions at matched time point. All *p* < 0.05. Sex‐specific post hoc analyses not performed if two‐way interaction was not significant (panels b, d). Bolded text indicates significance.

There was no condition × sex × time interaction (*p* = 0.254; Table S1) for relative FMD. There were condition × time (*p* = 0.002) and time × sex interactions (*p* = 0.016) for relative FMD. Post hoc analyses showed relative FMD was greater at 10 min post‐IRT, as compared to pre‐IRT (*p* = 0.025) and 40 min post‐IRT (*p* = 0.019) (Figure 2a). Further, relative FMD scores were higher for female than male participants at all time points (all *p* < 0.01; Figure 2b). There was no main effect of time (*p* = 0.397) nor a main effect of condition (*p* = 0.074). There were no between‐visit differences in FMD at pre‐intervention (*p* = 0.910) or 40 min post‐intervention (*p* = 0.673). However, FMD was greater 10 min post‐IRT relative to 10 min post‐rest (*p* < 0.001). Analyses were repeated on FMD values expressed as (1) absolute change, (2) allometrically scaled, and (3) shear normalized (Table 2, Table S1), with similar results.

**TABLE 2:** Artery characteristics.

	Condition	Pre	Post 10	Post 40	Condition × time *p*‐value
Baseline diameter (mm)	IRT	3.48 ± 0.65	3.43 ± 0.66	3.49 ± 0.66*	**0.021**
	Rest	3.52 ± 0.71	3.53 ± 0.66	3.49 ± 0.69	
Peak diameter (mm)	IRT	3.72 ± 0.62	3.72 ± 0.64	3.73 ± 0.67	0.306
	Rest	3.75 ± 0.69	3.72 ± 0.66	3.71 ± 0.71	
Absolute FMD (mm)	IRT	0.24 ± 0.10	0.29 ± 0.12	0.24 ± 0.11*	**0.002**
	Rest	0.23 ± 0.10	0.20 ± 0.10	0.22 ± 0.10	
Relative FMD (%)	IRT	7.33 ± 3.56	8.94 ± 4.42^a^	6.98 ± 3.46*	**0.002**
	Rest	7.00 ± 3.75	5.81 ± 3.26	6.55 ± 3.23	
Shear normalized FMD (%)	IRT	3.94 ± 1.48	4.82 ± 2.05^a^	3.82 ± 1.60*	**0.009**
	Rest	3.86 ± 1.55	3.39 ± 1.65	4.02 ± 1.89	
Scaled FMD (%)	IRT	7.02 ± 3.78	8.86 ± 4.16^a^	6.93 ± 3.28*	**0.003**
	Rest	6.94 ± 3.51	5.76 ± 3.09	6.50 ± 3.05	
Time to peak (s)	IRT	41.6 ± 8.8	40.8 ± 7.8	36.0 ± 11.0	0.754
	Rest	43.1 ± 11.4	40.2 ± 10.9	37.8 ± 8.9	
Baseline anterograde SR (s^−1^)	IRT	114.8 ± 29.9	109.2 ± 36.8	106.4 ± 30.3	0.695
	Rest	110.8 ± 33.9	104.0 ± 29.4	98.5 ± 26.4	
Hyperemia anterograde SR (s^−1^)	IRT	181.9 ± 46.1	183.3 ± 52.4	180.9 ± 47.5	0.090
	Rest	177.9 ± 43.9	171.0 ± 43.8	167.0 ± 39.7	
Hyperemic stimulus (%)	IRT	59.9 ± 17.8	72.3 ± 26.4	72.3 ± 20.8	0.104
	Rest	64.5 ± 17.9	65.8 ± 16.0	71.8 ± 21.3	
PWV (m/s)	IRT	6.99 ± 1.03	6.70 ± 0.76	6.86 ± 1.06	0.947
	Rest	6.90 ± 1.30	6.62 ± 1.08	6.84 ± 1.19	

*Note*: Data are mean ± SD. Two‐way repeated measures ANOVA; bold text indicates significant *p*‐value. There were no condition × sex × time interaction effects (Table S1). Hyperemic stimulus indicates the percent increase in shear rate during hyperemia, relative to baseline shear rate. Post hoc tests not performed if interaction effect was not significant.

Abbreviations: FMD, flow‐mediated dilation; IRT, inspiratory resistance training; post 10, 10 min after intervention end; post 40, 40 min after intervention end; pre, before intervention; PWV, pulse wave velocity; SR, shear rate.

^a^ Significantly different from baseline (post hoc).

* Significantly different from post 10 (post hoc); all *p* < 0.05.

There were no interaction effects on FMD‐related shear variables (all *p* > 0.05; Table 2, Table S1). There was a main effect of time on baseline (i.e., pre‐hyperemia) SR (*p* = 0.003). A follow‐up analysis showed baseline SR was lower 40 min post‐intervention, relative to pre‐intervention (*p* = 0.006), regardless of condition. There was also a main effect of time on the hyperemic stimulus (i.e., percent increase in SR induced by hyperemia; *p* = 0.004). A follow‐up analysis showed the hyperemic stimulus was lower at 40 min post‐intervention, relative to pre‐intervention (*p* = 0.025), regardless of condition.

### 3.3 Pulse wave velocity

There were no condition × sex × time (*p* = 0.625), condition × time (*p* = 0.947), nor condition × sex (*p* = 0.508) interaction effects on PWV values (Figure 2c). There was a main effect of time (*p =* 0.015), and follow‐up analyses showed PWV was slower 10 min post‐intervention, relative to pre‐intervention, regardless of condition. There was also a time × sex interaction (*p =* 0.006), wherein PWV was slower in males 10 min post‐intervention relative to pre‐ (*p* = 0.001) and 40 m post‐intervention (*p* = 0.003), regardless of condition (Figure 2d). No differences were noted in PWV between time points in female participants (all *p* > 0.05), nor were there between‐day differences in PWV in either sex (all *p* > 0.05).

### 3.4 Brachial artery diameter and SR patterns during IRT

**FIGURE 3:**
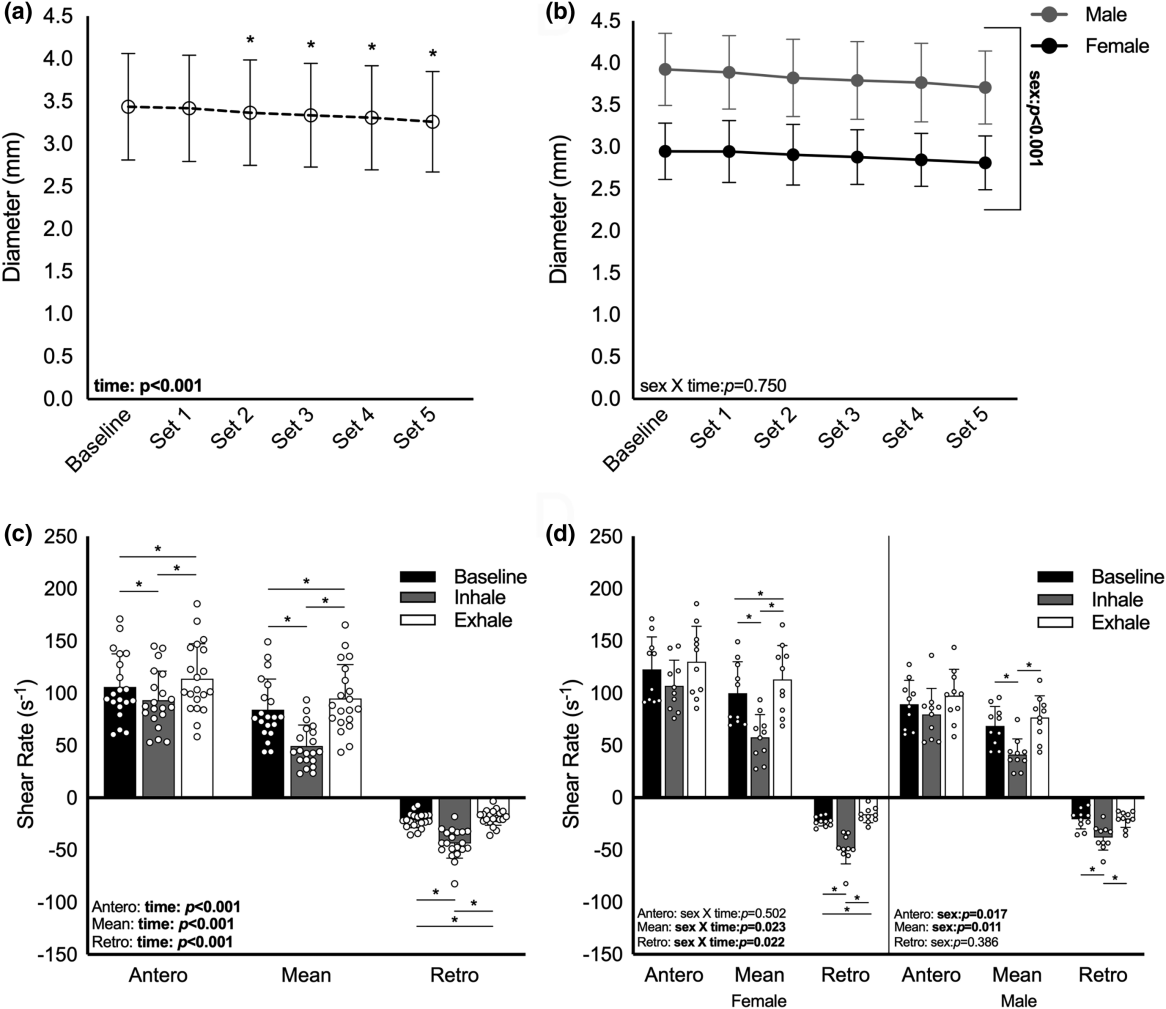
(a) Change in brachial artery diameter in response to IRT in healthy young men and women (a) combined (*n* = 20) and (b) separated by sex (10 male, 10 female). Repeated measures ANOVA. Post hoc analyses: *main effect of time, significantly different from baseline, *p* < 0.05. Post hoc tests not performed on panel b (sex × time interaction not significant). Differences in anterograde, mean, and retrograde SRs at baseline (black), during inspiration (gray), and during expiration (white) with men and women (c) combined (*n* = 20) and (d) separated by sex (10 male, 10 female). Repeated measures ANOVA, post hoc: *p < 0.05. Post hoc tests not performed on Anterograde SR in panel d (sex × time interaction not significant). Bolded text indicates significance.

We report a main effect of time on brachial artery diameter during IRT (*p* < 0.001; Figure 3a) but no sex × time interaction (*p* = 0.750; Figure 3b). Follow‐up analyses showed a reduction in artery diameter from baseline that reached significance at IRT set #2 (*p* = 0.023), and diameter continued to decline until the training bout was complete (Figure 3a). Compared to baseline, anterograde SR was lower during inspiration (*p* = 0.002) and higher during expiration (*p* = 0.012; Figure 3c, Table S2). There was no time × sex interaction (*p* = 0.502; Table S3).

Compared to baseline, retrograde SR was greater during inspiration (*p* < 0.001) and lower during expiration (*p* = 0.046) (Figure 3c). There was a significant time × sex interaction (*p* = 0.022), with post hoc analysis showing declines in retrograde SR during expiration, relative to baseline, in females (*p* = 0.003) but not in males (*p* = 0.999; Figure 3d). Retrograde SR was greater, relative to baseline, during inspiration for both males and females (*p* < 0.001).

Relative to baseline, mean SR was lower during inspiration (*p* < 0.001) and greater during expiration (*p* = 0.002; Figure 3c). A significant time × sex interaction (*p* = 0.023) highlighted greater mean SR during expiration, relative to baseline, for females (*p* = 0.007) but not males (*p* = 0.129). Mean SR during inspiration was lower than baseline in males and females (*p* < 0.001; Figure 3d).

### 3.5 Correlation between SR and change in flow‐mediated dilation

Compared to baseline, brachial artery FMD was greater 10 min post‐IRT (i.e., an IRT‐induced FMD enhancement). Pearson correlations showed a moderate positive correlation between retrograde SR during inspiration and IRT‐induced FMD enhancement (*r* = 0.437, *p* = 0.054). No other shear variables were associated with IRT‐induced FMD enhancement (all *p* > 0.10).

## 4 DISCUSSION

The purpose of this study was to determine the acute effects of a single bout of IRT on endothelial function and arterial stiffness and to characterize the SRs in the brachial artery during IRT. We report improved brachial artery FMD at 10 min post‐IRT that returns to baseline by 40 min post‐IRT. Additionally, we report a 102% increase in the magnitude of retrograde SR and a 10% decline in the magnitude of anterograde SR during resisted inspiratory efforts. By comparison, SRs during unresisted expiration differ only slightly from baseline (Figure 3c). Notably, the magnitude of improvement in FMD between pre‐IRT and 10 min post‐IRT was moderately associated with the magnitude of retrograde SR during the resisted inspiration phase (*r* = 0.437). This IRT‐related pulsatile retrograde shear stimulus may contribute to the process of beneficial vascular remodeling, however, additional research is needed to confirm this hypothesis. Despite several differences in baseline characteristics, men and women responded similarly to a single bout of IRT.

**FIGURE 4:**
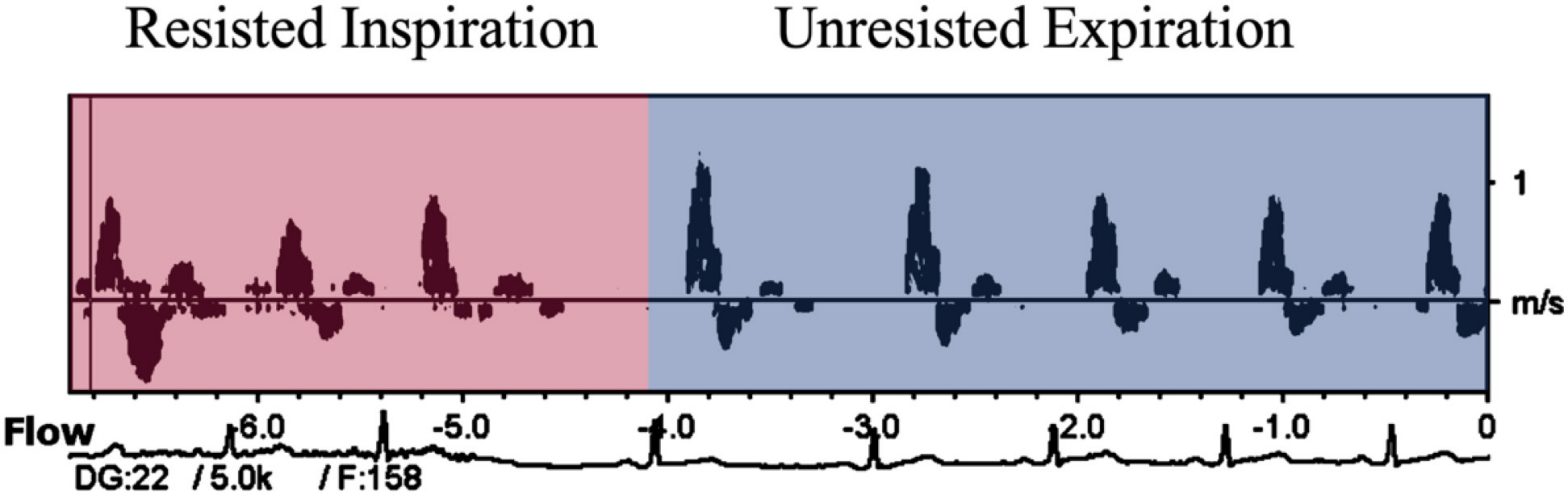
Ultrasound doppler of blood velocity during resisted inspiration (pink) and unresisted expiration (blue).

Exercise‐related increases in anterograde and/or mean SR are believed to be the primary mechanism underlying acute and long‐term improvements in FMD (Tinken et al., 2010). Whereas exercise‐induced SRs do not vary substantially beat‐to‐beat, the present findings highlight IRT‐related flow patterns that vary considerably beat‐to‐beat. The oscillatory shear patterns seen during IRT presumably are the result of large negative intrathoracic pressure swings drawing blood back to the heart through the arteries with each inspiratory effort. The effect of the intermittent “thoracic vacuum” during the inspiratory phase of the breath cycle effectively doubles retrograde SR for 1–2 heart beats per breath, but which returns to normal/baseline levels during the subsequent unresisted expiration (Figure 4). As IRT comprises a total of 30 such inspiratory efforts, the retrograde flow stimulus is rather brief.

A review of the literature on the impact of traditional exercise strategies on SRs yields mixed findings. Numerous studies indicate increased anterograde and mean SR (~2–4‐fold greater than baseline) were associated with acute enhancements in FMD (Johnson et al., 2012; Lyall et al., 2019; Tinken et al., 2009, 2010), while increased retrograde SR via blood flow restriction acutely impaired FMD (Thijssen et al., 2009). However, the latter is not a universal finding. For example, studies using enhanced external counterpulsation (EECP), a treatment for patients with coronary artery disease consisting of intermittent compression of the lower limbs during diastole, show increased retrograde SRs to supraphysiological levels without a substantial increase in anterograde SR, yet FMD is enhanced (Gurovich & Braith, 2013). Chronically high levels of retrograde SR during rest is a known risk factor for the development and progression of atherosclerosis, due in part to increased expression of proatherogenic genes, resulting in upregulation of endothelin‐1, superoxides and reactive oxygen species, and adhesion molecules (Chappell et al., 1998; De Keulenaer et al., 1998; McNally et al., 2003; Ziegler et al., 1998). Whereas both IRT and EECP contribute to acute and long‐term improvements in FMD (Braith et al., 2010; Craighead et al., 2021), it is unclear what effects these abbreviated interventions may exert on pro‐ and antiatherogenic mechanisms.

Previously, we showed progressive reductions in muscle sympathetic nerve activity during a single bout of IRT (DeLucia et al., 2021), a change typically associated with vasodilation. However, in the current study we observed a stepwise vasoconstriction during IRT (Figure 3a). Key differences between the studies, including body position (supine vs. seated), IRT intensity (50% vs. 75%), and measurement location (sympathetic nerve recordings from the lower leg vs. ultrasound imaging of the upper arm), may have contributed to the discrepant outcomes. Simultaneous recording of MSNA and ultrasound imaging of the brachial artery is needed to further elucidate the mechanisms underlying IRT‐induced vascular adaptations.

Although previous studies in healthy young adults show acute reductions in muscle sympathetic nerve activity during IRT (DeLucia et al., 2021), we found no evidence that the protocol impacts arterial stiffness. Our findings are in line with previous work showing 6 weeks of IRT has no impact on arterial stiffness in midlife and older adults with above‐normal blood pressure (Craighead et al., 2021).

While peak diameter following hyperemia was not different from pre‐IRT or 40 min post‐IRT, there was a persistent, albeit non‐significant, vasoconstriction in the period immediately post‐IRT (Table 2). This post‐IRT vasoconstriction resulted in absolute and relative FMDs that were greater at 10 min post‐IRT than at the other two time points. Gurovich and Braith (2013) reported similar findings in response to EECP, showing the acute enhancement in FMD could be attributed to post‐treatment reductions in baseline artery diameter while peak diameter remained unchanged. In comparison, a single bout of aerobic exercise resulted in a greater peak diameter following hyperemia, while baseline diameter was unchanged (Lyall et al., 2019; Tinken et al., 2010). It is possible that flow patterns unique to IRT and EECP elicit peripheral artery constriction via distinct mechanisms. Indeed, Mullen et al. (2001) demonstrated that FMD can be mediated by, or independent of, nitric oxide, based on the characteristics of the flow stimulus. How nitric oxide and shear‐mediated dilation are affected by IRT versus aerobic exercise warrants further investigation.

Despite differences in anthropometric and physiological outcomes at baseline (Table 1), both men and women responded similarly to IRT, with one exception. During the resisted inspiration phase of IRT, men and women exhibited similar changes in SR. However, during the unresisted expirations, SRs were significantly different from baseline in women but not men (Figure 3d). These differences were relatively small and did not appear to have a measurable effect on the acute FMD response to IRT.

### 4.1 Limitations

Our standardized IRT protocol is performed seated or standing with inspiratory resistance set at 75% of PI_MAX_. In the current study, our participants performed IRT supine at 50% PI_MAX_, thus, it is possible that the SRs reported here may differ from the standard protocol. Plouffe et al. (2023) reported similar changes in SRs in response to IRT at 15% and 75% of PI_MAX_, thus, we consider it unlikely that SRs would differ substantially as a function of relative intensity. Participants in the current study were healthy young adults naïve to IRT and performed training in supine, which alters respiratory mechanics (Lorino et al., 1992). Accordingly, we set the resistance to 50% PI_MAX_ to ensure the training was completed successfully. We cannot exclude the possibility that IRT may have different effects on FMD and SRs when performed in upright by older adults with endothelial dysfunction, as has been shown with traditional exercise (Green et al., 2017). This could be addressed in a future investigation.

We did not experimentally manipulate SR, therefore, we cannot state that the IRT‐related increases in retrograde SR is the stimulus underlying the transient improvement in FMD. Further research will be needed to differentiate between the effects of IRT on SR and other stimuli (e.g., release of exerkines from the respiratory musculature) that could induce improvements in endothelial function. We did not control for menstrual cycle phase of female participants in an effort to make the results more generalizable (Stanhewicz & Wong, 2020), and it is possible that our study was impacted by this decision. Finally, blood pressure was not assessed continuously during IRT in the current study. However, we have previously shown acute increases in blood pressure during and after IRT (DeLucia et al., 2021), which supports our finding of IRT‐induced vasoconstriction.

## 5 CONCLUSIONS

Our results show that retrograde SR is amplified during the resisted inspiration phase of IRT, and that there is a modest relationship between the magnitude of retrograde SR and FMD enhancement immediately following IRT. Further, a single bout of IRT induces progressive vasoconstriction of the brachial artery, with a residual constriction that persists for at least 3 min following IRT. IRT has no acute effects on arterial stiffness. Our findings may contribute to a better understanding of retrograde SR and how different types of shear stress (e.g., intermittent vs. continuous) may impact endothelial function and the regulation of pro‐ and antiatherogenic mechanisms. The acute effects of IRT were largely consistent between healthy men and women.

## AUTHOR CONTRIBUTIONS

All authors conceived of and designed the research. DT and JLM performed experiments. DT analyzed data, interpreted results, and drafted the manuscript. All authors edited, revised, and approved the final manuscript.

## FUNDING INFORMATION

This work was supported by NIA/NIH Grant Number: 1R01AG065346‐01A1 (EFB), NIH Training Grant Number: 5T32HL007249‐44 (DT), and NIH/NHLBI Grant Number: 1K01HL153326‐01 (DHC).

## CONFLICT OF INTEREST STATEMENT

The authors have no conflicts of interest to report.

## ETHICS STATEMENT

The procedures followed were in accordance with the ethical standards of the University of Arizona IRB and with the Helsinki Declaration of 1975, as revised in 2008.

## Supporting information


Table S1.
Table S2.
Table S3.

## Data Availability

The data that support the findings of this study are available from the authors but restrictions apply to the availability of these data. Data are, however, available from the authors upon reasonable request.
