# Supplementary material for: Acute inspiratory resistance training enhances endothelium‐dependent dilation and retrograde shear rate in healthy young adults
Source: Physiol Rep. 2024 Feb 4;12(3):e15943. doi: 10.14814/phy2.15943 (PMC10838658; doi:10.14814/phy2.15943)
Supplement: Supplementary file 1 — Table S1. Table S2. Table S3. [file PHY2-12-e15943-s001.docx]

**Acute inspiratory resistance training enhances endothelium-dependent dilation and retrograde shear rate in healthy young adults**

Dallin Tavoian, PhD; Josie L. Mazzone, MS; Daniel H. Craighead, PhD; E. Fiona Bailey, PhD

**Supplemental Tables**

|  |  | Female | | |  | Male | | | Condition x Sex x Time |
| --- | --- | --- | --- | --- | --- | --- | --- | --- | --- |
|  |  | Pre | Post 10 | Post 40 |  | Pre | Post 10 | Post 40 | *p*-value |
| Baseline Diameter (mm) | IRT | 2.99 ± 0.35 | 2.93 ± 0.38 | 3.00 ± 0.39 |  | 3.97 ± 0.48 | 3.92 ± 0.50 | 3.98 ± 0.49 | 0.550 |
|  | Rest | 3.00 ± 0.44 | 3.04 ± 0.42 | 2.99 ± 0.44 |  | 4.04 ± 0.51 | 4.02 ± 0.47 | 3.99 ± 0.51 | - |
| Peak Diameter (mm) | IRT | 3.28 ± 0.38 | 3.28 ± 0.39 | 3.26 ± 0.47 |  | 4.15 ± 0.50 | 4.15 ± 0.55 | 4.19 ± 0.52 | 0.956 |
|  | Rest | 3.29 ± 0.47 | 3.26 ± 0.47 | 3.22 ± 0.47 |  | 4.22 ± 0.54 | 4.19 ± 0.49 | 4.20 ± 0.55 | - |
| Absolute FMD (mm) | IRT | 0.30 ± 0.07 | 0.35 ± 0.08 | 0.26 ± 0.11 |  | 0.19 ± 0.09 | 0.23 ± 0.14 | 0.22 ± 0.12 | 0.740 |
|  | Rest | 0.28 ± 0.10 | 0.22 ± 0.11 | 0.23 ± 0.12 |  | 0.18 ± 0.07 | 0.17 ± 0.09 | 0.21 ± 0.08 | - |
| Relative FMD (%) | IRT | 9.98 ± 2.40 | 11.96 ± 3.11 | 8.52 ± 3.53 |  | 4.69 ± 2.34 | 5.93 ± 3.38 | 5.44 ± 2.75 | 0.254 |
|  | Rest | 9.55 ± 3.52 | 7.31 ± 3.60 | 7.79 ± 3.96 |  | 4.46 ± 1.69 | 4.30 ± 2.11 | 5.31 ± 1.70 | - |
| Shear Normalized FMD (%) | IRT | 4.84 ± 1.13 | 5.69 ± 1.29 | 4.04 ± 1.53 |  | 3.03 ± 1.24 | 3.94 ± 2.34 | 3.59 ± 1.71 | 0.501 |
|  | Rest | 4.75 ± 1.41 | 3.79 ± 1.81 | 4.23 ± 2.14 |  | 2.97 ± 1.16 | 2.99 ± 1.45 | 3.81 ± 1.70 | - |
| Scaled FMD (%) | IRT | 9.95 ± 2.20 | 11.92 ± 2.82 | 8.47 ± 3.33 |  | 4.15 ± 2.85 | 5.88 ± 3.25 | 5.41 ± 2.64 | 0.285 |
|  | Rest | 9.50 ± 3.24 | 7.25 ± 3.39 | 7.73 ± 3.74 |  | 4.44 ± 1.64 | 4.28 ± 2.04 | 5.29 ± 1.63 | - |
| Time to peak (s) | IRT | 42.1 ± 8.9 | 40.8 ± 8.5 | 34.2 ± 13.6 |  | 41.0 ± 9.2 | 40.7 ± 7.6 | 37.8 ± 8.0 | 0.977 |
|  | Rest | 45.6 ± 13.3 | 42.9 ± 12.9 | 38.8 ± 10.8 |  | 40.5 ± 9.0 | 37.5 ± 8.3 | 36.9 ± 6.9 | - |
| Baseline Anterograde SR (s^-1^) | IRT | 130.5 ± 25.8 | 128.6 ± 37.9 | 119.2 ± 31.5 |  | 99.2 ± 26.0 | 89.8 ± 24.3 | 93.5 ± 24.1 | 0.176 |
|  | Rest | 126.9 ± 39.4 | 117.2 ± 33.9 | 110.0 ± 30.5 |  | 93.3 ± 15.8 | 90.9 ± 16.9 | 87.1 ± 15.8 | - |
| Hyperemia Anterograde SR (s^-1^) | IRT | 210.4 ± 42.3 | 214.6 ± 53.3 | 209.9 ± 45.4 |  | 153.5 ± 30.0 | 152.1 ± 29.9 | 151.8 ± 28.5 | 0.937 |
|  | Rest | 201.2 ± 47.0 | 195.9 ± 46.7 | 189.4 ± 41.9 |  | 154.5 ± 25.5 | 146.2 ± 22.2 | 144.7 ± 21.6 | - |
| Hyperemic Stimulus (%) | IRT | 61.5 ± 12.0 | 70.0 ± 22.3 | 78.7 ± 19.8 |  | 58.3 ± 22.7 | 74.4 ± 30.9 | 66.0 ± 20.7 | 0.144 |
|  | Rest | 62.7 ± 20.1 | 69.3 ± 15.9 | 75.4 ± 21.5 |  | 66.4 ± 16.2 | 62.2 ± 16.1 | 68.3 ± 21.7 | - |
| PWV (m/s) | IRT | 6.44 ± 1.01 | 6.36 ± 0.69 | 6.37 ± 0.80 |  | 7.59 ± 0.66 | 7.09 ± 0.68 | 7.41 ± 1.08 | 0.625 |
|  | Rest | 6.22 ± 0.81 | 6.34 ± 1.04 | 6.18 ± 0.84 |  | 7.64 ± 1.37 | 6.94 ± 1.10 | 7.58 ± 1.11 | - |

**Supplemental Table 1. Artery characteristics split by sex**. Data are mean ± SEM. Two-way repeated measures ANOVA. Post hoc tests not performed if interaction effect was not significant. FMD, flow-mediated dilation; IRT, inspiratory resistance training; Post 10, 10 minutes after intervention end; Post 40, 40 minutes after intervention end; Pre, before intervention; PWV, pulse wave velocity; SR, shear rate

|  | Pre-IRT | Inhale | Exhale | *p*-value |
| --- | --- | --- | --- | --- |
| Anterograde SR (s^-1^) | 105.9 ± 31.6 | 93.2 ± 27.9* | 113.8 ± 33.5*^†^ | **<0.001** |
| Mean SR (s^-1^) | 84.2 ± 29.2 | 49.4 ± 20.0* | 94.8 ± 32.4*^†^ | **<0.001** |
| Retro SR (s^-1^) | 21.7 ± 7.2 | 43.8 ± 14.0* | 19.0 ± 7.4*^†^ | **<0.001** |

**Supplemental Table 2. Shear rate during IRT.** Data are mean ± SEM. Repeated measures ANOVA; bold text indicates significant p-value. *Significantly different from baseline (post hoc); ^†^Significantly different from Post 10 (post hoc); all *p*-values <0.05. SR, shear rate

|  | Female | | |  | Male | | | Sex x Time |
| --- | --- | --- | --- | --- | --- | --- | --- | --- |
|  | Baseline | Inhale | Exhale |  | Baseline | Inhale | Exhale | *p*-value |
| Anterograde SR (s^-1^) | 122.5 ± 31.3 | 106.9 ± 24.5 | 130.1 ± 33.9 |  | 89.3 ± 22.8 | 79.5 ± 25.0 | 97.5 ± 25.2 | 0.502 |
| Mean SR (s^-1^) | 99.9 ± 30.2 | 57.6 ± 21.8* | 113.0 ± 32.4*^†^ |  | 68.6 ± 18.6 | 41.2 ± 15.0* | 76.6 ± 20.8^†^ | **0.027** |
| Retro SR (s^-1^) | 22.6 ± 4.6 | 49.3 ± 14.1* | 17.0 ± 7.0*^†^ |  | 20.7 ± 9.3 | 38.3 ± 12.0* | 20.9 ± 7.6^†^ | **0.022** |

**Supplemental Table 3. Shear rate during IRT split by sex.** Data are mean ± SEM. Two-way repeated measures ANOVA; bold text indicates significant p-value. *Significantly different from baseline (post hoc); ^†^Significantly different from Post 10 (post hoc); all *p*-values <0.05. Post hoc tests not performed if interaction effect was not significant. SR, shear rate
